# Biocompatibility and Antimicrobial Profile of Acid Usnic-Loaded Electrospun Recycled Polyethylene Terephthalate (PET)—Magnetite Nanofibers

**DOI:** 10.3390/polym15153282

**Published:** 2023-08-02

**Authors:** Alexandra Elena Stoica (Oprea), Alexandra Catalina Bîrcă, Dan Eduard Mihaiescu, Alexandru Mihai Grumezescu, Anton Ficai, Hildegard Herman, Baltă Cornel, Marcel Roșu, Sami Gharbia, Alina Maria Holban, Bogdan Ștefan Vasile, Ecaterina Andronescu, Anca Oana Hermenean

**Affiliations:** 1 Department of Science and Engineering of Oxide Materials and Nanomaterials, University Politehnica of Bucharest, 060042 Bucharest, Romania; oprea.elena19@gmail.com (A.E.S.); grumezescu@yahoo.com (A.M.G.); ecaterina.andronescu@upb.ro (E.A.); 2 Department of Organic Chemistry, University Politehnica of Bucharest, 011061 Bucharest, Romania; danedmih@gmail.com; 3 ICUB—Research Institute of the University of Bucharest, 060102 Bucharest, Romania; 4 Academy of Romanian Scientists, Ilfov No. 3, 050044 Bucharest, Romania; 5 Institute of Life Sciences, Vasile Goldis Western University of Arad, 310414 Arad, Romania; herman.hildegard@uvvg.ro (H.H.); samithgh2@hotmail.com (S.G.); hermenean.anca@uvvg.ro (A.O.H.); 6 Microbiology Immunology Department, Faculty of Biology, University of Bucharest, 030018 Bucharest, Romania; alina_m_h@yahoo.com; 7 National Research Center for Micro and Nanomaterials, University Politehnica of Bucharest, 060042 Bucharest, Romania; bogdan.vasile@upb.ro; 8 Research Center for Advanced Materials, Products and Processes, University Politehnica of Bucharest, 060042 Bucharest, Romania

**Keywords:** recycled PET, magnetite, usnic acid, electrospinning, nanofibers, antimicrobial agents, in vitro, in vivo, biocompatibility

## Abstract

The highest amount of the world’s polyethylene terephthalate (PET) is designated for fiber production (more than 60%) and food packaging (30%) and it is one of the major polluting polymers. Although there is a great interest in recycling PET-based materials, a large amount of unrecycled material is derived mostly from the food and textile industries. The aim of this study was to obtain and characterize nanostructured membranes with fibrillar consistency based on recycled PET and nanoparticles (Fe_3_O_4_@UA) using the electrospinning technique. The obtained fibers limit microbial colonization and the development of biofilms. Such fibers could significantly impact modern food packaging and the design of improved textile fibers with antimicrobial effects and good biocompatibility. In conclusion, this study suggests an alternative for PET recycling and further applies it in the development of antimicrobial biomaterials.

## 1. Introduction

Nanofibers have emerged as novel nanostructured materials with wide applicability [1] in numerous applications, including filtration [2], tissue engineering [3,4], biosensors [5,6], wound dressing [7,8], nanofibrous composites [9], protective clothing [10], food packaging [11], and drug delivery systems [12,13]. There are various techniques for nanofiber development, including electrospinning, phase separation, self-assembly, freeze-drying, template synthesis, the spinneret-based tunable engineered parameter method, and interfacial nanofiber polymerization [14]. Electrospinning (ES) represent a versatile and straightforward technique widely used to obtain continuous fibers from a large number of polymers, with diameters ranging from tens of nanometers to several micrometers [15,16]. The resulting fibrous mats have a large effective surface area, continuously interconnected pores (usually with high and controllable porosities) and high surface roughness [17].

Polyethylene terephthalate (PET) is a transparent and easy-to-process polymer, often utilized for food and beverage packaging [18]. Additionally, physico-chemical properties highlight PET as an ideal candidate for the design of shoes, clothing, bedding, and interior materials for automobiles [19]. Hence, there is a continuously increasing interest in the application of PET for medical purposes, such as artificial blood vessels, artificial heart valves, hernia repair meshes and scaffolds, and sewing rings [20,21,22].

As the third most commonly exploited polymer [13], the total global consumption of PET is around 13 million tons annually, mostly for fabricating packaging and textile fibers [23]. With an estimated production of 34 billion metric tons by 2050 [24], there is an increasing concern for the environment, which led many scientists to consider using recycled PET for different applications.

Nano-sized materials have superior physical and chemical properties compared to their bulk counterparts due to their mesoscopic, small object, quantum size, and surface effects [25,26]. Specifically, magnetite nanoparticles’ characteristics, including non-toxicity, biocompatibility, and super-paramagnetism [27], make them ideal for biomedical application [28]. In this context, magnetite nanoparticles have been widely investigated in dynamic sealing [29], ecosystems [30], magnetic resonance imaging as contrast agents [31], therapeutic hyperthermia [32], biosensing [33,34], and magnetic targeted-drug delivery [35,36]. Moreover, magnetite nanoparticles have proved their efficiency in antimicrobial therapies due to their intrinsic antimicrobial properties and the capacity to deliver antimicrobial agents [37]. In this regard, nano-systems comprising magnetite nanoparticles functionalized with usnic acid, a lichen secondary metabolic compound with proven antimicrobial, antibiotic, and tissue regeneration capacities [38], represent a promising alternative for antimicrobial therapies. Moreover, recent studies have focused on incorporating magnetite-based bioactive materials containing antimicrobial agents or microorganism colonization inhibitors into nanostructured polymeric membranes. In recent decades, such approaches have significantly impacted their potential to overcome the challenges associated with biofilm formation and antimicrobial resistance.

This study aimed to develop and characterize nanostructured membranes with fibrillar consistency based on recycled PET and magnetite nanoparticles functionalized with usnic acid by electrospinning technique. To the best of our knowledge, no available studies report the direct synthesis of iron oxide nanoparticles onto the nanofibers’ surface, as most use blends comprising PET and iron oxide nanoparticles for electrospinning. Moreover, the available studies do not investigate the biocompatibility or antimicrobial efficiency of the obtained biomaterials. Thus, we presumed to design a highly efficient antimicrobial biomaterial offering a potential alternative for recycling PET. The synthesized membranes’ antimicrobial activity were assessed against *S. aureus*, *P. aeruginosa*, and *C. albicans*, in both planktonic and biofilm states, and the biocompatibility of the usnic acid- loaded electro-spun recycled PET nanofibers was assessed by in vitro and in vivo methods.

## 2. Materials and Methods

### 2.1. Materials

The polyester polymer was obtained from recycled PET coke bottles that were approved for the food industry. Dichloromethane (Mw = 84.96 g/mol) was acquired from Chimopar Trading SRL and trifluoroacetic acid (Mw = 114.02 g/mol) was purchased from Fluka Analytical. Ferrous sulfate 7-hydrate (FeSO_4_∙7H_2_O), ferric chloride (FeCl_3_), and ammonia (NH_3_, 25%) were purchased from Sigma-Aldrich (St. Louis, MO, USA). All chemicals were of analytical purity and used with no further purification.

### 2.2. Electrospinning Deposition of PET Nanofibers

The electrospinning (ES) technique was utilized to fabricate nanostructured mats from recycled PET, according to our previously published article [39]. This method has been used to obtain membranes consisting of fibrous networks with interconnected, overlapping, and randomly distributed fibers. First, the PET bottles were cut into small pieces (about 1 cm^2^) and then submerged in a mixture of dichloromethane and trifluoroacetic acid (volume ratio 1:8). The polymer was completely dissolved in the mixture, and electrospinning was performed using the parameters described in Table 1. The electrospinning procedure was carried out using a Tong Li Tech (Shenzhen, China) ES equipment, with a 23.26 kV voltage (−5.73 kV and 17.53 kV), 200 mm needle-to-target distance, and 5, 7.5 and 10 mL/h, respectively, flow rate for 30 min for all solutions.

### 2.3. Magnetite (Fe_3_O_4_) Functionalized with Usnic Acid (UA) Synthesis

The iron oxide nanoparticles were obtained by wet chemical precipitation from aqueous iron salt solutions using alkaline media.

The usnic acid-functionalized magnetic nanoparticles were prepared using wet chemical co-precipitation from aqueous iron salt solutions using alkaline media. Thus, a first solution of Fe^2+^ and Fe^3+^ in 1:2 molar ratio was prepared (300 mL) according to Refs. [40,41,42]. Then, a second solution was made using NH_4_OH solution (25%, 9 mL) and added to a 0.03% solution of usnic acid (300 mL).

### 2.4. Polyethylene Terephthalate (PET)—Magnetite Nanofibers Functionalized with Usnic Acid Synthesis

PET nanofibers obtained via electrospinning were cut into 1 cm^2^ pieces and submerged in the first solution for 10 min (described in Section 2.3). After that, they were submerged in the second solution for another 10 min (described in Section 2.3). Subsequently, the samples were washed with distilled water and left to dry at room temperature overnight. Thus, depending on the feed rate of the electrospinning, three types of samples were obtained and noted accordingly (PET@Fe_3_O_4_@UA_5, PET@Fe_3_O_4_@UA_7.5, and PET@Fe_3_O_4_@UA_10).

### 2.5. Physico-Chemical Characterization

#### 2.5.1. Fourier-Transform Infrared Spectroscopy

IR spectra were obtained with a Nicolet iN10 MX Fourier-transform (FT-IR) microscope from Thermo Fischer Scientific (Waltham, MA, USA) equipped with a liquid nitrogen-cooled mercury cadmium telluride (MCT) detector. The spectral collection was registered in reflection mode at a resolution of 4 cm^−1^ in the 700–4000 cm^−1^ wavenumber range, and 32 scans were co-added for each spectrum and converted to absorbance using the OmincPicta software (version 8.2 Thermo Nicolet) from Thermo Scientific.

#### 2.5.2. X-ray Diffraction (XRD)

Grazing incidence X-ray Diffraction (GIXRD) was investigated with a PANalytical Empyrean diffractometer (PANalytical, Almelo, The Netherlands) utilizing CuK radiation (=1.541874 A) equipped with a 2 × Ge (2 2 0) hybrid monochromator for Cu and a parallel plate collimator on the PIXcel3D. With a step size of 0.04° and a time for each step of 3 s, scanning was carried out on the 2θ axis in the range of 5–80° with an incidence angle of 0.5°.

#### 2.5.3. Scanning Electron Microscopy

The morphology and size of the fiber mats were carried out by Scanning Electron Microscopy using equipment purchased from FEI (Hillsboro, OR, USA). The samples were cut with a diamond disc and fixed on a sample support for placement in the analysis chambre. The obtained images are obtained by recording the resultant secondary electron beam with 30 keV energy at different points of the samples.

#### 2.5.4. Transmission Electron Microscopy

In order to obtain important information on the inmate microstructure of fibrous mats, Transmission Electron Microscopy (TEM) images were acquired. The samples were fixed on a carbon-coated copper grid at room temperature (RT). Obtaining TEM images was possible by analyzing the sample using a high-resolution TecnaiTM G2 F30 S-TWIN transmission microscope equipped with SAED, purchased from Thermo Fisher Scientific (former FEI, Hillsboro, OR, USA). This equipment operates in transmission mode using 300 kV voltage, the point and line resolution guaranteed, with values of 2 Å and 1 Å, respectively.)

#### 2.5.5. FT-ICR-MALDI

The FT-ICR MALDI method involves positive ionization mode, 4 M data acquisition magnitude, 90–2500 uam mass range, 100 V plate offset voltage, 260 V deflector plate voltage, 25% laser power with 250 laser shots at 1500 Hz frequency and, for ion optics, 0.7 ms time of flight at 4 Mhz frequency, 350 Vpp RF amplitude.

### 2.6. Biological Characterization

#### 2.6.1. In Vitro Antibacterial Experiments

Growth of planktonic (free-floating) microorganisms in the presence of materials. To test the effect on planktonic microorganism growth, the obtained materials were cut into 1 cm/1 cm samples and then sterilized by exposure to UV radiation for 30 min on each side. One fragment of sterile material was individually deposited in a well of a sterile 6-well plate, 2 mL of nutritive broth was added to each well, and then 20 μL of 0.5 McFarland microbial suspension (*Staphylococcus Aureus ATCC 23235* and *Pseudomonas aeruginosa ATCC 25619*) or 1 McFarland (yeast—*Candida albicans*) prepared in sterile physiological water (0.9% NaCl solution). The 6-well plates were incubated at 37 °C for 24 h. After the incubation time expiration, 200 μL of the obtained microbial suspensions were transferred to 96 sterile plates, and the turbidity of the microbial cultures (absorbance) was measured spectrophotometrically at 600 nm.

Evaluation of adhesion and biofilm formation. To test the effect of fibrillated materials on adhesion and biofilm production, the materials were cut to 1 cm/1 cm and sterilized by exposure to UV radiation for 20 min on each side. One fragment of sterile material was individually deposited in a well of a 6-well sterile plate, and 2 mL of liquid medium and then 20 μL of 0.5 McFarland (bacteria—*S. aureus* and *P. aeruginosa*) or 1 McFarland (yeast—*C. albicans*) microbial suspension prepared in sterile physiological water were added to the wells. The plates were incubated at 37 °C for 24 h. After incubation, the materials were washed with sterile saline water and placed into the sterile nutritive broth. The samples were incubated for different periods (24, 48, and 72 h, respectively) to allow the development of attached cells and biofilm formation. After the expiration of each incubation period, the sample on which the biofilm was developed was washed with sterile saline water and placed in 1 mL of sterile saline water. The tube was vigorously vortexed for 30 s and sonicated for 10 s to separate the cells from the biofilm. The prepared cell suspension was diluted, and different dilutions were seeded on solid culture media plates to result in and quantify colony-forming units (CFU/mL). The statistical significance (* *p* ≤ 0.05, ** *p* < 0.001) was determined using the non-parametric two-way ANOVA algorithm Bonferroni test.

#### 2.6.2. In Vivo Experiments

Animals and experimental design. The in vivo experiments were performed after the approval of the protocol by the Research Ethics Commission of the Vasile Goldis Western University of Arad.

Experimental studies used adult CD1 mice housed in IVC cages with standard breeding conditions in the university’s animal facility.

The materials sterilized in UV light (30 min on each side) were implanted in a subcutaneous pocket in the dorsal region, under anesthesia, by intraperitoneal administration of xylazine/ketamine.

Seven experimental groups (*n* = 10) were performed, as follows: control, PET_5_ctrl, PET_7.5_ctrl, PET_10_ctrl, PET@Fe_3_O_4_@UA_5, PET@Fe_3_O_4_@UA_7.5, and PET@Fe_3_O_4_@UA_10, and euthanized after 24 h and 7 days after surgery.

After the surgery, the animals were housed individually and examined clinically every day by a vet, according to the following parameters: the appearance of surgery, redness, infection, edema/abscess, hematoma, and scars. Biopsies were performed 24 h, respectively, 7 days after implantation, under anesthesia. Blood was also collected by cardiac puncture for biochemical analysis.

Biochemistry. The collected blood was centrifuged at 3500 rpm for 10 min. Samples were analyzed for C-reactive protein (CRP) level evaluation on a Mindray BS-120 (ShenzenMindray Bio-Medical Electronics Co., Ltd., Nanshan, Shenzhen, China) chemistry analyzer, using the CRP FL reagent kit (ChemaDiagnostica, Monsano, Italy).

Histology. The surrounding tissue’s implant area was fixed in 4% paraformaldehyde solution, embedded in paraffin, sectioned at 5 μm, and stained with hematoxylin and eosin (H&E) and Masson Goldner trichrome. The microscopic sections were analyzed under the microscope (Olympus BX43 equipped with an Olympus XC30 digital camera and CellSens software V4.2, Shinjuku, Japan). Sections were scored to grade the inflammation, fibrosis, and neovascularization. Each histometric parameter was graded on a scale of 0–4 for the amount of tissue reaction: − (not present) to ++++ (extensive).

Immunofluorescence. Deparaffinization and rehydrated sections were exposed to primary antibody TNF-α (Abcam (Cambridge, UK), dilution 1:100) after antigen unmasking with sodium citrate buffer (pH 6.0) and BSA blocking for 1 h. Alexa Fluor dye conjugated (1:500) was used as a secondary antibody, and nuclei were counterstained with DAPI. The fluorescence was visualized by confocal microscopy (Leica TCS SP8 confocal microscope, Wetzlar, Germany).

## 3. Results and Discussions

The nanostructured membranes obtained by electrospinning and subsequently impregnated with magnetite nanoparticles functionalized with usnic acid have been characterized by FT-IR, XRD, SEM and TEM. Fe_3_O_4_@UA characterization has been presented elsewhere [41]. Furthermore, previous attempts have been made to develop magnetic nanofibers based on iron oxide nanoparticles and PET through the electrospinning method [43,44]. However, to the best of our knowledge, no available studies report the direct synthesis of iron oxide nanoparticles onto the nanofibers’ surface, as most use blends comprising PET and iron oxide nanoparticles for electrospinning. Moreover, the available studies do not investigate the biocompatibility or antimicrobial efficiency of the obtained biomaterials.

### 3.1. X-ray Diffraction

**Figure 1:**
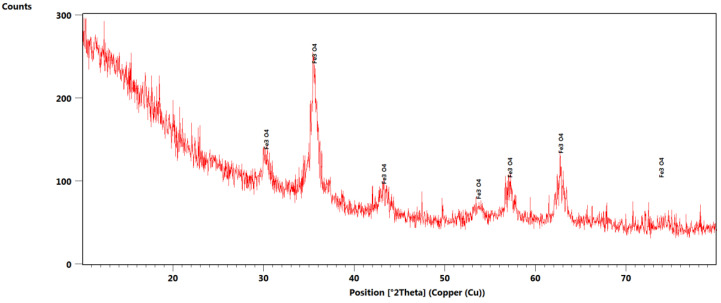
X-ray diffractogram recorded for PET@Fe_3_O_4_@UA.

Figure 1 shows the X-ray diffractogram recorded for PET@Fe_3_O_4_@UA. A single crystalline phase along with the diffractive interference characteristic of the magnetite is observed. However, the presence of PET resulted in a reduced crystallinity of the sample. The planes (220), (311), (400), (422), (511), (440) and (533) Bragg’s reflections attributed to 2θ angle: 30.2°, 35.6°, 43.2°, 53.7°, 57.2°, 62.8° and 74.3° correspond to the face-centered cubic (fcc) structures of magnetite, which are in accordance with JCPDS No. 79-0417 [45,46].

### 3.2. Scanning Electron Microscopy

**Figure 2:**
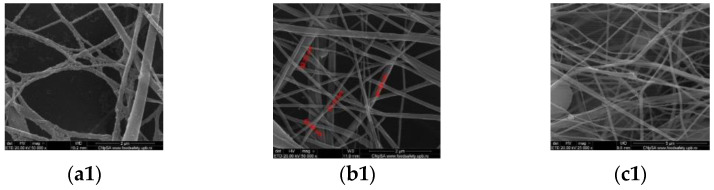

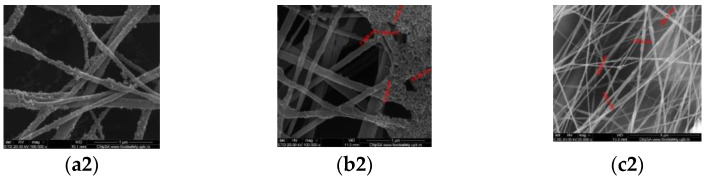
SEM images for PET@Fe_3_O_4_@UA at various flows ((**a1**,**a2**)—5 mL/h; (**b1**,**b2**)—7.5 mL/h; (**c1**,**c2**)—10 mL/h).

Scanning Electron Microscopy (SEM) was used to characterize the morphology of the nanostructured membranes obtained via electrospinning at various feed rates and to confirm the nanoparticles’ presence on the surface of the nanofibers. The results are shown in Figure 2. A nanostructured wire network with diameters ranging from 50 to 150 nm is observed for all experimental deposition rates. Furthermore, it can be seen that Fe_3_O_4_@UA are randomly distributed, usually at the junction of the fibers, which act as nucleation centers favoring the growth of magnetite nanocrystals. While the size of the nanofibers is smaller, the distribution of the nanoparticles is in accordance with our previous work [39]. Furthermore, the particle size present on the surface of the junction between the fibers varies between 5 and 10 nm.

Therefore, it can be assumed that the formation of nanoparticles directly onto the nanofibers’ surface does not affect their size, morphology, and properties. However, the tendency of the magnetite nanoparticles to form clusters at the nanofibers’ junction could further affect their antimicrobial potency due to a consequently reduced bioavailability [47] and to their mechanical properties [48]. The nanoparticle aggregation issue could be resolved by subjecting the solutions in which the PET meshes are immersed to magnetic stirring, thus ensuring a homogenous deposition of the iron precursors onto the nanofibers’ surface.

### 3.3. Transmission Electron Microscopy

**Figure 3:**
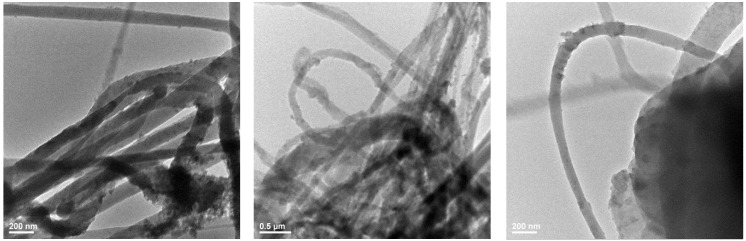
TEM images for nanostructured membranes of PET@Fe_3_O_4_@UA at a flow rate of 5 mL/h (PET@Fe_3_O_4_@UA_5).

TEM characterization further confirmed the results obtained through SEM analysis. TEM images are shown in Figure 3. Thus, the nanometric diameter of the fibers with sizes between 50 and 150 nm is confirmed, with a non-homogenous distribution of the nanoparticles onto their surface. Results from previous studies focusing on the development of PET nanofibers through the electrospinning method report significantly higher nanofiber sizes [49,50].

### 3.4. Fourier-Transform Infrared Spectroscopy

**Figure 4:**
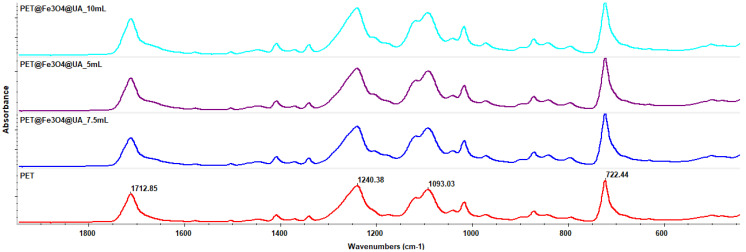
The FT-IR spectra for PET@Fe_3_O_4_@UA membranes.

FT-IR was used to assess the integrity of functional groups after the electrospinning process. The results obtained are shown in Figure 4 and highlight the presence of PET-absorbing bands, namely 1712 cm^−1^ and 1240 cm^−1^, characteristic for the C=O of the ester group and absorption bands for the asymmetrical C-O-C stretching and C-H aromatic ring bonds at 1093 cm^−1^ and 722 cm^−1^, respectively [51,52,53]. Additionally, the absorption band at 1017 cm^−1^ is characteristic of the in-plane vibration of benzene [54]. No movement of the absorption bands is observed. The Fe-O bond absorption band characteristic for magnetite is not observed. The amount of magnetite nanoparticles was under the detection limit of diamond-ATR crystal.

### 3.5. FT-ICR MALDI

**Figure 5:**
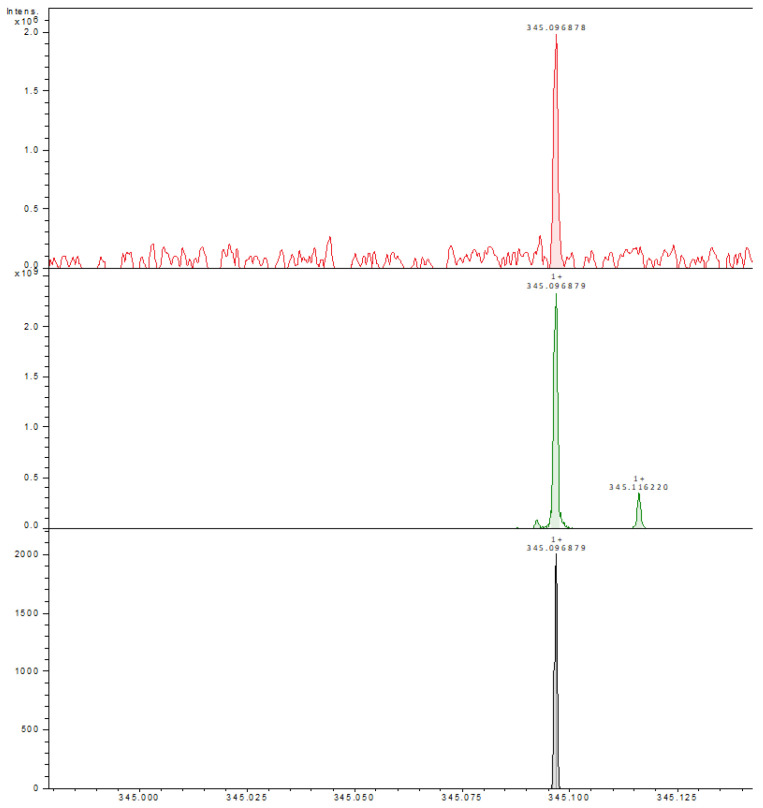
Zoom-in from the full spectrum (upside) to the monoisotopic peak of usnic acid, sample (red), reference (green), and simulated spectrum (black).

**Figure 6:**
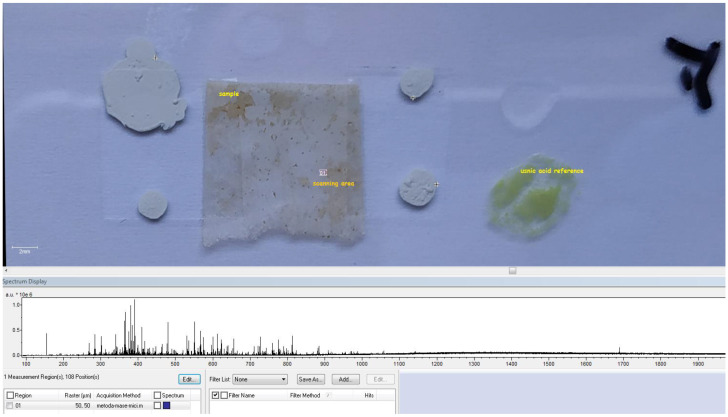
MALDI slide, sample (PET@Fe_3_O_4_@UA), reference compound, and scanning area.

The usnic acid identification was performed by HR-FT-ICR-MS method using the MALDI sample introduction technique and DHB (di-hydroxibenzoic acid) matrix. As a mass reference compound, high-purity usnic acid was used (middle spectrum in figure—green) and the mass confirmation was performed by Compass DataAnalysis mass cluster simulator (black spectrum, down). The sample mass peak (red spectrum, upside) proves a low mass difference from the usnic acid reference at a 288,625 FWHW mass resolution, allowing a positive identification of usnic acid in the thin film sample (Figure 5). The sample preparation method involves sample and usnic acid reference immobilization on an ITO-grafted slide and DHB matrix (methanolic solution) deposition (nebulization) before FT-ICR analysis (Figure 6).

**Figure 7:**
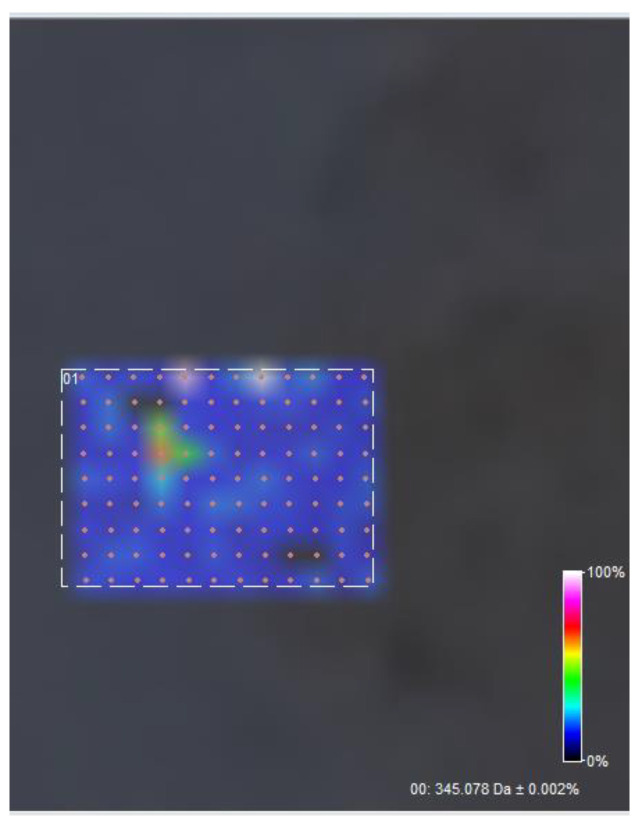
Surface distribution of usnic acid M+H peak, C18H16O7.

**Figure 8:**
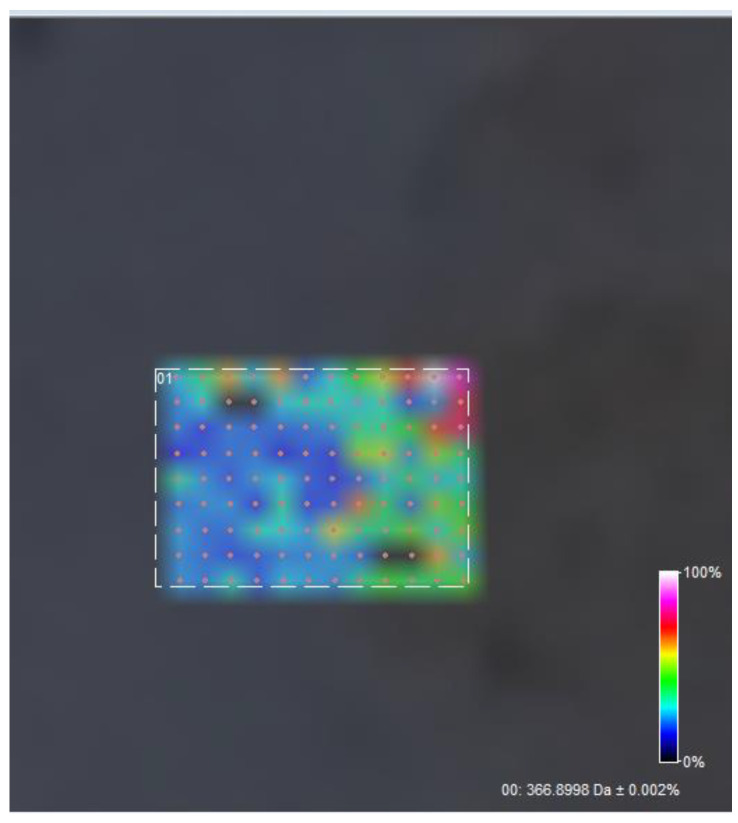
Surface distribution of usnic acid M+Na peak, C18H16O7Na.

A further surface scan of the sample (108 data acquisition points, 50 × 50 um spacing) reveals a relative homogeneous surface distribution of usnic acid in the scanned area (Figure 7 and Figure 8).

### 3.6. In Vitro Biocompatibility

#### Antimicrobial Effect

Contamination of foods and medical surfaces with pathogenic microorganisms represents a significant risk factor for consumers and patients. Microorganisms can grow both in free-floating (planktonic) cultures and attached to surfaces by producing highly specialized multicellular communities called biofilms. Adherent microorganisms have different biochemical and genetic traits and represent an additional risk factor, as they are more difficult to remove and more resistant than microorganisms that develop in a planktonic state. Biofilm bacteria show behavior-related resistance to antimicrobials and host defense mechanisms, which differs from genetically acquired microbial resistance, and is known as tolerance. In this context, alternative methods for limiting microbial colonization and biofilm formation are being intensively studied for industrial and medical purposes [55,56].

**Figure 9:**
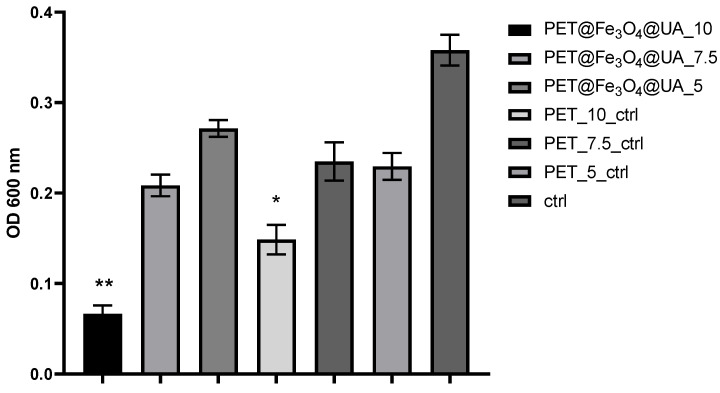
Graphical representation of the recorded absorbance values for *S. aureus* cultures, expressing the multiplication capacity of these cells after cultivation for 24 h in the presence of recycled PET polymer materials and control (planktonic microorganisms without materials). * *p* < 0.05; ** *p* < 0.001.

For *S. aureus* planktonic cultures (Figure 9), it was observed that recycled PET containing F_3_O_4_@UA nanoparticles had a significant inhibitory effect against microbial growth. It can be observed that the highest inhibitory activities were achieved in PET samples at which the fibers deposition by electrospinning was realized at a flow rate of 10 mL/h, followed by the samples obtained at a flow of 7.5 mL/h.

**Figure 10:**
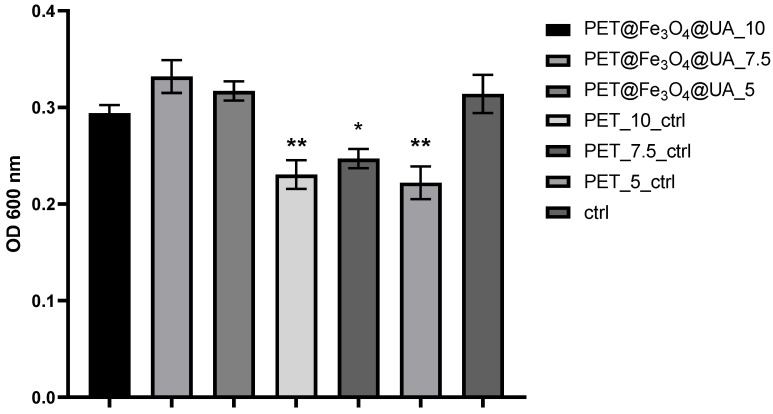
Graphical representation of the absorbance values recorded for *P. aeruginosa* cultures expressing the multiplication capacity of bacteria cells after cultivation for 24 h in the presence of recycled PET polymer materials and control (planktonic microorganisms without materials). * *p* < 0.05; ** *p* < 0.001.

In the case of planktonic *P. aeruginosa* cultures, it was also observed that PET@Fe_3_O_4_@UA exhibited good inhibition against microbial development. Compared with the results observed for *S. aureus* cultures, *P. aeruginosa* growth inhibition is frequently lower in all experimental variants (Figure 10).

**Figure 11:**
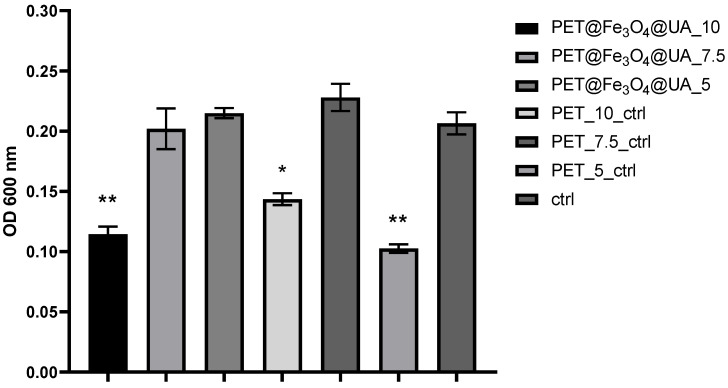
Graphical representation of the absorbance values recorded for cultures of *C. albicans*, expressing the multiplication capacity of these cells after cultivation for 24 h in the presence of recycled PET polymer materials and control (planktonic microorganisms without materials). * *p* < 0.05; ** *p* < 0.001.

In the case of the tested yeast strain, it can be observed that the effect of the obtained materials on the growth of planktonic *C. albicans* is relatively uniform for the samples obtained at a flow rate of 7.5 and 5 mL/h, which are similar to the control. However, growth is significantly inhibited by the sample obtained at a 10 mL/h flow rate (Figure 11).

The assessment of biofilm formation capacity proved different results than those obtained in planktonic cultures, suggesting that attachment inhibition and biofilm modulation may be a specific effect of these materials.

**Figure 12:**
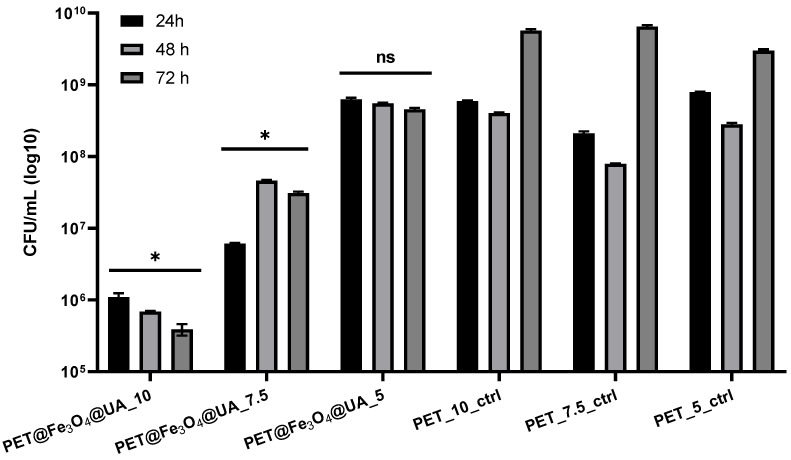
Graphical representation of CFU/mL (colony forming units/mL) representing the number of *S. aureus* cells included in the monospecific biofilms developed on the surface of the materials obtained for 24 h, 48 h, and 72 h at 37 °C. (* *p* < 0.05; by comparing biofilm formation on PET control and corresponding UA containing PET, ns = not significant).

The inhibition effect of *S. aureus* biofilm development was achieved at all stages of biofilm development, starting with the cell adherence phase (up to 24 h), continuing with the maturation stage (up to 48 h) and until dispersion (when cells or cell aggregates detach from biofilm to colonize new surfaces) (Figure 12).

PET@Fe_3_O_4_@UA has also demonstrated an effect of inhibiting the growth of biofilms produced by *S. aureus* at all time intervals analyzed, with the highest efficiency for the 5 mL/h samples.

**Figure 13:**
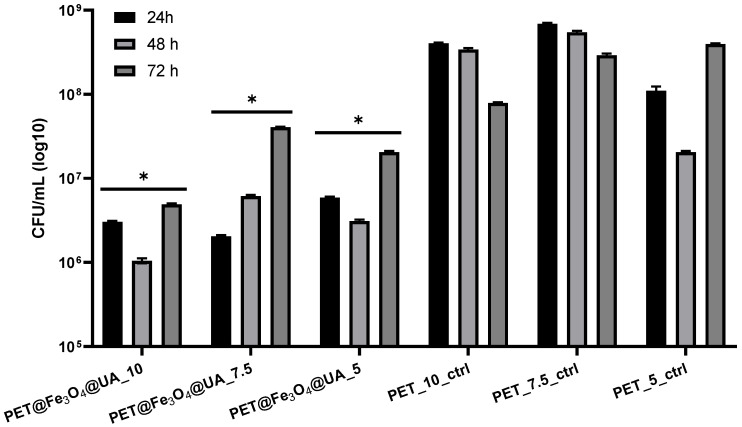
Graphical representation of CFU/mL (colony forming units/mL) representing the amount of *P. aeruginosa* cells included in the monospecific biofilms developed on the surface of the materials obtained for 24 h, 48 h, and 72 h at 37 °C. (* *p* < 0.05; by comparing biofilm formation on PET control and corresponding UA containing PET).

*P. aeruginosa* is a bacterial species with various natural resistance mechanisms, being an opportunistic pathogen that can colonize and adhere efficiently in various environments. Biofilms produced by *P. aeruginosa* are difficult to eradicate with actual antimicrobial medicines [57]. The results presented in our study have shown that *P. aeruginosa* presents a limited ability to form biofilms onto the obtained nanostructured fibrous mats (Figure 13).

The obtained PET@Fe_3_O_4_@UA membranes produced significant inhibition of *P. aeruginosa* biofilms at all tested time intervals, regardless of the fiber flow rate deposition by electrospinning.

**Figure 14:**
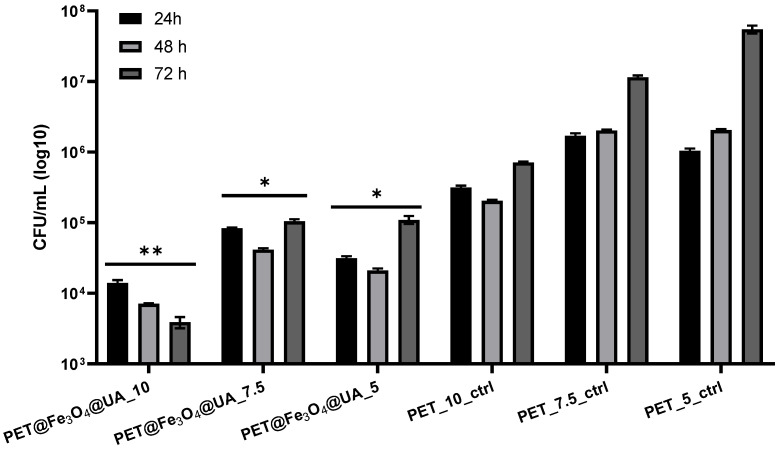
Graphic representation of CFU/mL (colony forming units/mL) representing the number of *C. albicans* cells included in the monospecific biofilms developed on the surface of the materials obtained for 24 h, 48 h, and 72 h at 37 °C. (* *p* < 0.05; ** *p* < 0.001 by comparing biofilm formation on PET control and corresponding UA containing PET).

In the case of the *C. albicans* strain, significant biofilm development inhibition capacities can be observed in all experimental variants tested. All membranes based on PET and inorganic nanoparticles have shown an inhibitory effect on the development of *C. albicans* biofilms, regardless of the fiber deposition rate by electrospinning or the type of nano-system contained, and not being influenced by the action time (Figure 14).

While most studies focus on the antimicrobial effects of silver and gold nanoparticles, this study provides evidence of the efficiency of magnetite nanoparticles obtained onto the surface of PET nanofibers against microbial growth and colonization, still limited in the literature. The precise mechanisms involved could be associated with nanoparticles’ properties in terms of reduced size and high surface-to-volume ratios, and surface reactivity [58].

Moreover, since the concentration of magnetite nanoparticles was not modified, the differences in the obtained samples’ antimicrobial activity are influenced by the parameters applied in the electrospinning process. Precisely, the best results were obtained for the samples prepared with a feed rate of 10 mL/h in the case of *S. aureus* and *C. albicans*, both in planktonic and biofilm states. Reports from the available literature state that the feed rate parameter does not influence nanofibers’ diameter [59,60]. As PET reported no intrinsic antimicrobial properties, the increased antimicrobial activity of the 10 mL/h samples could be associated with a higher number of magnetite nanoparticles formed onto the surface of the nanofibers.

Furthermore, results prove a higher efficiency of the obtained biomaterials against Gram-positive and yeast species than Gram-negative bacteria, possibly due to the differences in their structural features.

**Figure 15:**
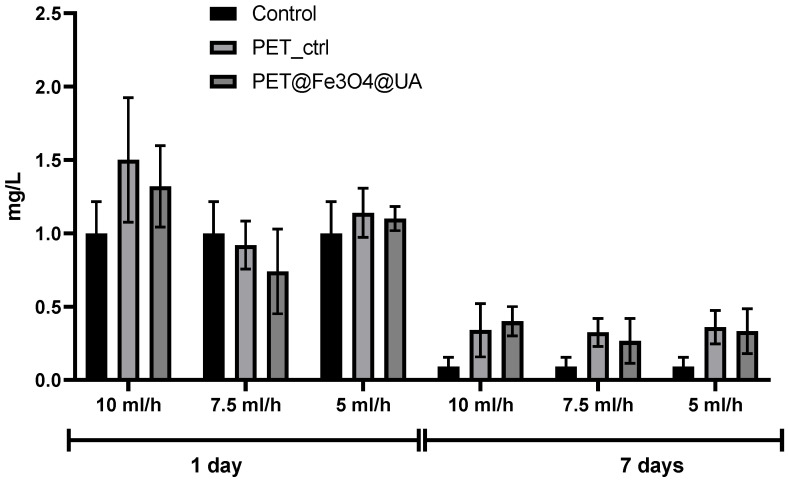
The effects of PET@Fe_3_O_4_@UA subcutaneous implantation in mice on the C-reactive protein (CRP) levels at 24 h and 7 days post-surgery.

Figure 15 shows the effects of the subcutaneous implantation of PET@Fe_3_O_4_ on the serum level of the inflammatory marker CRP. At 24 h after implantation, serum CRP concentration increased for all experimental groups, followed by a 14-day decrease. The CRP level was decreased for PET@Fe_3_O_4_ implants compared to the PET control group.

**Figure 16:**
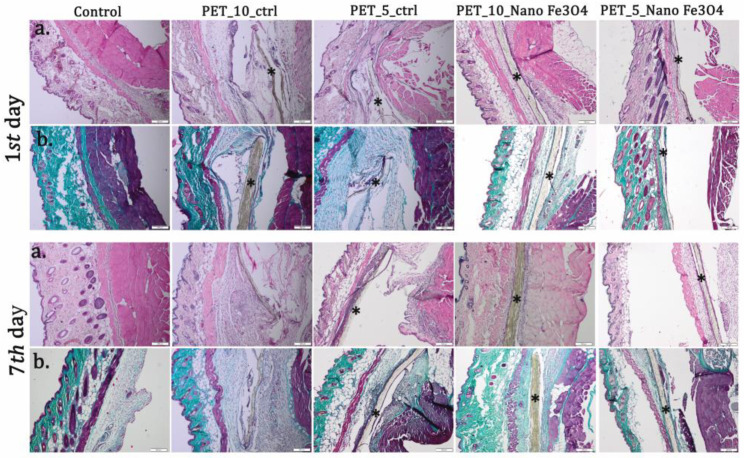
Biocompatibility analysis of PET_Fe_3_O_4_@UA_10 and PET@Fe_3_O_4_@UA_5 at 24 h and 7 days post-implantation. (**a**) H&E stain; (**b**) Masson-Goldner trichrome stain. Material (*); Scale bar 200 μm.

The post-implantation clinical analysis revealed no local or systemic adverse effects. Histopathological analysis showed edema in the case of PET control at 24 h, which is maintained at 7 days after surgery (Figure 16). The inflammatory reaction is strongest for PET control at 10 mL/h. The leukocyte count in the tissues surrounding these implants revealed the marked presence of PMN at 24 h, a sign of acute inflammation. At 7 days, they are replaced gradually with macrophages (Table 2). At this interval, the presence of fibroblasts and collagen deposits is noticed, highlighted by trichrome stain (Figure 16). The inflammatory reaction is much reduced in the case of PET coating with Fe_3_O_4_ nanoparticles functionalized with usnic acid, which decreased with the flow rate. Moreover, the repair process is highlighted, demonstrated by the newly formed capillaries in the damaged tissues around the implant (Table 2).

**Figure 17:**
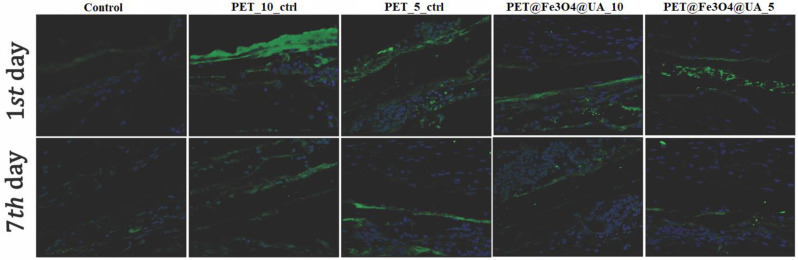
TNF-α protein expression as revealed by confocal microscopy at 24 h and 7 days post-implantation. TNF-α is labeled green, and the nuclei are counterstained with DAPI.

Immunofluorescence was performed for tissue sections to analyze inflammatory response towards the implanted materials. As shown in Figure 17, TNF-α immunostaining increased on soft tissue surrounded by PET-materials with flow rate. The immunoreaction was decreased, though PET was coated with Fe_3_O_4_.

The rapid advancement of technology and nanoscience, along with the rapid dissemination of innovative findings, has allowed the development of nanofibers obtained via electrospinning. It is essential to note that this method differs from others because it allows for controlling of the diameter, morphology, orientation, and even fiber structure [61].

There are studies on the electrospinning of PET, but only a few published papers regarding the electrospinning of recycled PET, where electrospinning from melt was used to produce fibers in the nanometer–few micrometer range, with most applications in smoke or air filtration [61,62,63] or oil-water separation [64] and only a very small number concerning application in anti-infective therapy [39]. It is essential to keep in mind that the research into recycled materials has attracted an increased interest in the past decade, owing to the necessity to minimize waste and develop alternative sources of resources [61].

Voltage, ambient temperature, humidity, heat power, feed rate, and needle-to-target distance were all carried out thoroughly during the electrospinning process described in the present work. Optimal electrospinning conditions were achieved using a trial-and-error method at −5.73 kV, +17.53 kV, and 0.6 kW with different feed rates under ambient conditions of 35% relative humidity and a temperature of 27 °C. However, in the process of obtaining (nano)fibrous membrane using the ES technique, the voltage crucial because no fiber can be formed because the surface tension of the solution prevents the solution from flowing into the collector. A constant needle-to-target distance of 200 mm was maintained for all samples, given that the distance influences the fibers’ diameter. To achieve that goal, one of the most important parameters of the electrospinning process was varied—rate deposition (feed rate—5 mL/h, 7.5 mL/h, and 10 mL/h)—and their influence on fiber morphology, biocompatibility and antimicrobial and antibiofilm activity against Gram-positive and Gram-negative bacteria strains, but also on opportunistic yeast, was analyzed. The morphology of PET fibers was examined by scanning electron microscopy, Transmission Electron Microscopy, Fourier-Transform Infrared Spectroscopy, and X-ray Diffraction. A nanostructured fibrous mat with diameters ranging from 50 to 150 nm is noticed for all experimental feed rates according to SEM images. Moreover, we can establish that Fe_3_O_4_@UA are randomly distributed on the samples, frequently at the junction of the fibers, which are considered as nucleation centers promoting the growth of magnetite nanocrystals. Besides, the particle size observed on the surface of the junction between the fibers ranges from 5 to 10 nm. These results were also confirmed by Transmission Electron Microscopy. Moreover, these fibrillar nanoparticle-containing membranes showed good inhibition in vitro due to usnic acid-functionalized magnetic nanoparticles presenting a remarkably enhanced antimicrobial activity against both Gram-positive (*S. aureus*) and Gram-negative (*P. aeruginosa*) bacteria strains, but also on opportunistic yeast *C. albicans*, as compared to control. The functionalized fibrous mat also showed low toxicity in vivo, and clinical analysis performed post-implantation revealed no local or systemic adverse effects.

Thus, the findings presented in this study open up new possibilities for PET recycling, such as combining it with other antimicrobial inorganic nanostructures to create enhanced fibrillar materials with antimicrobial and antibiofilm capabilities. Such technologies could be used in the food business, particularly for food packing, as well as in the biomedical field, to generate antimicrobial medical fabrics.

## 4. Conclusions

The obtained PET nanostructured membranes showed an improved antimicrobial and antibiofilm activity against model Gram-positive (*S. aureus*) and Gram-negative (*P. aeruginosa*) bacteria strains, but also on opportunistic yeast *C. albicans*. The best results in terms of antimicrobial potential were obtained for the samples obtained at higher feed rates due to the formation of denser meshes and with higher amounts of magnetite nanoparticles on their surface (qualitative observation). Moreover, these fibrillar nanoparticle-containing membranes showed low toxicity in vitro and in vivo. The results open new perspectives for PET recycling, such as its use combined with various antimicrobial inorganic nanostructures to obtain improved fibrillar materials with antimicrobial and antibiofilm properties. Such systems could be further utilized in the food industry, especially for food packaging applications, but also in the biomedical field to develop antimicrobial medical textiles.

## Figures and Tables

**Table 1:** The parameters used for electrospinning.

Sample	Output 1 (kV)	Output 2 (kV)	Heat (kW)	Humidity (%)	Temperature (°C)	Feed Rate (mL/h)
PET_5_ctrl	−5.73	17.53	0.6	35	27	5
PET_7.5_ctrl						7.5
PET_10_ctrl						10

**Table 2:** Histomorphometric scoring used to grade inflammation and neovascularization in the tissue surrounding subcutaneous implants.

Material	Implantation Period (Days)	Edema	PMN	M	F	NV
Control	1	−	+	−	−	−
	7	−	−	+	−	−
PET_10_ctrl	1	++++	+++	++	+	−
	7	+++	++	+++	++++	−
PET_7.5_ctrl	1	+++	+++	+	+	−
	7	++	+	+++	+++	−
PET_5_ctrl	1	++	+++	+	+	−
	7	++	+	+++	++	+
PET@Fe_3_O_4_@UA_10	1	+	++	+	+	−
	7	−	+	++	+++	++
PET@Fe_3_O_4_@UA_7.5	1	−	+	+	+	−
	7	−	−	++	++	++
PET@Fe_3_O_4_@UA_5	1	−	+	−	+	−
	7	−	−	++	++	++

PMN: Polymorphonuclear neutrophils; M: Macrophages; F: Fibroblasts, NV: Neovascularization; Tissue reactions are rated from − (not present) until ++++ (extensive).

## Data Availability

Available at the authors, at request.
